# Analysis of Iron Oxide Reduction Kinetics in the Nanometric Scale Using Hydrogen

**DOI:** 10.3390/nano10071276

**Published:** 2020-06-30

**Authors:** Swathi K. Manchili, Johan Wendel, Eduard Hryha, Lars Nyborg

**Affiliations:** Department of Industrial and Materials Science, Chalmers University of Technology, 41296 Gothenburg, Sweden; johan.wendel@chalmers.se (J.W.); hryha@chalmers.se (E.H.); lars.nyborg@chalmers.se (L.N.)

**Keywords:** nanopowder, conversion factor, activation energy, thermogravimetry

## Abstract

Iron nanopowder could be used as a sintering aid to water-atomised steel powder to improve the sintered density of metallurgical (PM) compacts. For the sintering process to be efficient, the inevitable surface oxide on the nanopowder must be reduced at least in part to facilitate its sintering aid effect. While appreciable research has been conducted in the domain of oxide reduction of the normal ferrous powder, the same cannot be said about the nanometric counterpart. The reaction kinetics for the reduction of surface oxide of iron nanopowder in hydrogen was therefore investigated using nonisothermal thermogravimetric (TG) measurements. The activation energy values were determined from the TG data using both isoconversional Kissinger–Akahira–Sunose (KAS) method and the Kissinger approach. The values obtained were well within the range of reported data. The reaction kinetics of Fe_2_O_3_ as a reference material was also depicted and the reduction of this oxide proceeds in two sequential stages. The first stage corresponds to the reduction of Fe_2_O_3_ to Fe_3_O_4_, while the second stage corresponds to a complete reduction of oxide to metallic Fe. The activation energy variation over the reduction process was observed and a model was proposed to understand the reduction of surface iron oxide of iron nanopowder.

## 1. Introduction

### 1.1. Background

The use of metal nanopowder instead of the conventional micrometre-sized powder for the powder metallurgical process route in order to manufacture near fully dense material has been of great interest for high performance applications [1]. The use of nanopowder however reduces the green density of conventionally die-pressed compacts owing to poor compressibility. Hence, the increased sintering activity will then necessarily not be enough to bring the material into required densification. One approach would then be the use of it as an additive to conventional powder to improve the densification within the conventional press and sinter route to at least facilitate closed porosity, which would open new opportunities, e.g., post-sintering capsule-free hot isostatic pressing. During the sintering of such powder mix, i.e., conventional micrometre sized powder and nanopowder additive, the nanopowder sinters at low temperatures to initiate early onset of sinter necks and also contributes to overall densification [2]. Sintering, being a surface phenomenon, implies that it is important to understand the surface constituents in order to design a sintering process that is efficient.

Nanoscale materials are of interest owing to their modified solid-state properties compared to conventional solids [3]. Nanosized metal powder are being produced and extensively used for a wide range of applications. The principal interest stems from the properties that nanomaterials possess owing to their excess surface energy. This excess surface energy contributes to the extraordinary chemical characteristics like melting point depression and reduced activation energy for oxide reduction [4,5]. Deviation in the melting temperature is observed with an increased proportion of surface atoms when the particle size is reduced beyond a critical value [6], typically about 10 nm.

Iron nanopowder is hence used in the present research as an additive to improve the density of water-atomized iron powder compacts. The iron nanopowder was found to be covered with an oxide scale of 3 nm thick [7]. For the sintering process to be efficient, this surface oxide on the metal particle must at least in part be reduced. The reduction of surface oxide is a necessary must for the metallurgical bonds to form between the metal particles comprising the compact; which in turn is crucial for achieving the structural strength of the PM component. This study aims at understanding the reaction kinetics for reduction of the aforementioned oxide scale on the iron nanopowder when heated in pure hydrogen. Investigation on the reduction kinetics of the micron meter sized powder has already been studied [8]. Iron nanopowder of two different size fractions was investigated and thermogravimetry was applied to depict the reduction kinetics. Two different size fractions were studied as this study is part of a larger effort to understand how nanopowder enhances densification in water atomised steel powder. Then, the activation energy (E) was evaluated using two different approaches, namely the isoconversional Kissinger–Akahira–Sunose (KAS) method and Kissinger approach. A similar exercise was carried out on Fe_2_O_3_ reference material to develop a systematic understanding of the reduction kinetics for the presumed main constituent of the oxide layer. The study hence aims to clarify the governing fundamental factors for the reduction of the surface oxide layer.

### 1.2. Theory

The methods used for kinetic analysis can be categorized based on experimental conditions and mathematical analysis used. Mathematical models are further divided into model fitting and isoconversional methods. Isoconversional methods are known as model-free methods. In the case of nonisothermal studies, model fitting methods are not preferred due to various reasons. They assume that the kinetic parameters like pre-exponential parameter (A), activation energy (E) are constant and use a single heating rate curve to fit the parameters [9]. Activation energy and pre-exponential factor are assumed to remain constant during solid-state reactions but they seem to vary as the reaction progresses [10]. Isoconversional methods are used to investigate this variation. Isoconversional methods have become popular in comparison to the model fitting methods. They are based on data from several experiments, and the reaction rate at a particular degree of conversion is considered to be a function of temperature. The model-free kinetics analysis uses this variation for evaluating the reaction mechanism but the disadvantage with this approach is that the pre-exponential factor cannot be directly evaluated. A series of experiments at different heating rates are to be conducted to be able to use isoconversional methods [11].

The calculations for the apparent activation energy in this study have been performed using nonisothermal experimental data. In this study, activation energy is calculated using two different approaches, namely the isoconversional and Kissinger approaches.

In a gas/solid reaction, the basic reaction considered is gas reacting with solid yielding a product. The rate of reaction, which is the change in the concentration of the reactants or the products over the change in time, can be described according to the following equation:

(1) $$Rate\;=\frac{-\left[gas\right]}{dt}=k{\left[gas\right]}^{n}$$

where [gas] is the gas concentration, n is the order of the reaction, and k is the rate constant.

When the rate constant k relates with temperature through activation energy, it is given in the form of Arrhenius equation:

(2) $$k=Ae^{\frac{-E}{RT}}$$

where E is the activation energy, R is the universal gas constant, A is frequency or pre-exponential factor, and T is temperature.

The progress or extent of the reaction or the conversion fraction of a reaction is given by α as:

(3) $$\alpha =\frac{m_{i}-m}{m_{i}-m_{f}}$$

where $m_{i}$ is the initial mass, m is the actual mass, and $m_{f}$ is the final mass of the sample under kinetic study.

The reaction rate can be described as:

(4) $$\frac{d\alpha }{dt}=kf\left(\alpha \right)$$

where f(α) is the function to explain the reaction model. The above equation is integrated for integral rate law according to:

(5) $$\int_{0}^{\alpha }\frac{d\alpha }{f\left(\alpha \right)}=g\left(\alpha \right)=kt$$

where g(α) is the function to explain the integration reaction model. Based on f(α) and g(α), the solid–gas reaction models, namely Avrami–Erofeev model, unimolecular model, 1-D, 2-D, and 3-D models, and power law model, are proposed [12].

Combining Equations (2) and (5), we get:

(6) $$g\left(\alpha \right)=Ae^{-E/RT}t$$

Expressing the reaction rate as a function of temperature for nonisothermal systems is done by using the expression as follows:

(7) $$\frac{d\alpha }{dT}=\frac{d\alpha }{dt}\;\frac{dt}{dT}$$

where $\frac{dT}{dt}$ is the heating rate represented by *β*. Thus, the Equation (7) above can be rewritten as:

(8) $$\frac{d\alpha }{dT}=\frac{kf\left(\alpha \right)}{\beta }$$

Using Equation (2), the Equation (8) can be further rewritten as:

(9) $$\frac{d\alpha }{dT}=\frac{A}{\beta }e^{-E/RT}f\left(\alpha \right)$$

Integrating Equation (9) yields:

$$\int_{0}^{\alpha }\frac{d\alpha }{f\left(\alpha \right)}=\frac{A}{\beta }\int_{T_{o}}^{T}e^{-E/RT}dT$$

(10) $$g\left(\alpha \right)=\frac{A}{\beta }\int_{T_{o}}^{T}e^{-E/RT}dT$$

This equation can be rewritten as:

(11) $$g\left(\alpha \right)=\frac{AE}{\beta R}\;p\left(x\right)$$

where $x=\frac{E}{RT}$ and p(x) is the exponential integral.

There are different isoconversional methods to evaluate E at different degrees of conversion, α. The Ozawa, Flynn and Wall method uses a plot of logβ verses $\frac{1}{T}$ plot whose slope gives E for a particular α [13]. There are other methods like the Friedman method and the nonlinear Vyazovkin method which have their own set of advantages or disadvantages [14,15].

This study used the Kissinger–Akahira–Sunose (KAS) method, for which Equation (9) can be integrated and rearranged as follows:

(12) $$\ln\frac{\beta }{T^{2}}=-\frac{E}{RT}+\ln\left(\frac{AE}{g\left(\alpha \right)R}\right)$$

where the term $\ln\left(\frac{AE}{g\left(\alpha \right)R}\right)$ is independent of T and β. For a particular α, the slope of the plot $\ln\frac{\beta }{T^{2}}$ versus $\frac{1}{T}$ obtained at different heating rates yields the activation energy.

#### The Kissinger Method

The Kissinger method is based on a series of experiments in which the sample is heated at different heating rates (β), whereby the activation energy can be determined from the correlation between maximum mass loss at so called peak temperature. The peak temperature, $T_{m}$, for the reduction reaction is measured for each heating rate. This peak temperature is hence when the mass loss rate is the highest. A plot is constructed of $\ln\left(\frac{\beta }{T_{m}^{2}}\right)$ versus $\frac{1}{T_{m}}$ and a linear relation is fitted to the data. The slope obtained is $\frac{-E}{R}$ where E is the activation energy and R is the gas constant [16]. This approach was used to derive the activation energy for the reduction of both the Fe nanopowder and the Fe_2_O_3_ reference nanopowder.

## 2. Materials and Methods 

Iron nanopowder of two different size fractions, namely 40–60 and 60–80 nm, was acquired from Sigma-Aldrich, Germany. The purity of the metal powder was higher than 99.5 wt. %. The reference Fe_2_O_3_ nanopowder was also obtained from the same source. The size of the oxide powder was less than 50 nm. The morphology of both metal and oxide powder was spherical.

X-ray photoelectron spectroscopy (XPS) was carried out using a PHI 5000 Versaprobe III (Physical Electronics Inc, Chanhassen, MN, USA) X-ray photoelectron spectrometer equipped with monochromatic AlK_α_ (1486.6 eV) X-ray source. The XPS was carried out to know the chemistry of oxide scale on the iron nanopowder. Narrow scans were carried out using 69 eV pass energy and the data analysis was performed using MultiPak software, version 9.7 ((Physical Electronics Inc, Chanhassen, MN, USA). X-ray diffraction was performed on the oxide nanopowder using a Bruker AXS D8 Advance diffractometer (Karlsruhe, Germany) with CrK_α_ radiation operated at 35 kV and 50 mA. The scanning range of 30–158° with a step size of 0.02° at 2.5 sec/step was employed.

To investigate the reduction kinetics, thermogravimetric analysis was performed using a simultaneous thermal analyzer STA449 (NETZSCH Thermal Analysis GmbH, Germany). A small alumina crucible was used as a vessel for the sample to be analyzed. Crucible along with the sample was placed in the middle of the vertical furnace on top a thermocouple pole. Before the experiment, the furnace was evacuated and then flushed with argon gas. The mass of the samples used for thermogravimetric analysis (TG) was 100 ± 10 mg. The powder sample was heated from room temperature to 1000 K under high purity hydrogen atmosphere (99.9999% purity) at various heating rates of 5, 7, 10, 15, 20, 25, 30, 40, and 50 K/min. Constant flow rate of 100 mL/min of the high purity hydrogen gas was employed. Scientific purity argon gas was used as protective gas at a constant flow rate of 20 mL/min. Mass loss is recorded as a function of temperature under controlled atmosphere. As described in the theory section, the activation energy for the hydrogen-driven reduction of the oxide layer on the Fe nanopowder and the Fe_2_O_3_ was calculated by plotting the obtained data from TG curves in an Arrhenius relation based on the estimation procedure described in the calculation of reduction kinetics section above.

## 3. Results and Analysis

### 3.1. Iron Nanopowder

#### 3.1.1. Non-Isothermal Reduction of Oxide Layer on Fe Nanopowder

**Figure 1:**
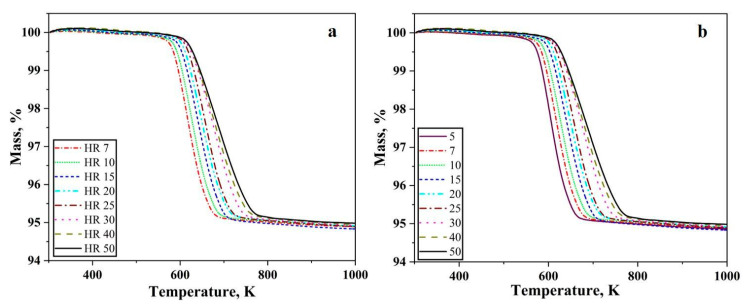
TG graphs for the reduction of oxide layer on Fe nanopowder in pure hydrogen up to 1000 K, showing results for size ranges (**a**) 40–60 and (**b**) 60–80 nm, respectively. The numbers in the inserts show the different heating rates applied in K/min.

Figure 1 shows the thermogravimetry results for hydrogen-driven reduction of oxide layer on the iron nanopowder. Figure 1a,b shows the heating stage of the reduction for the iron nanopowder size range of 40–60 nm and 60–80 nm, respectively, at different heating rates.

A total loss of around 5% was recorded independent on heating under all the conditions for both the nanopowder. The reduction process commences around 573 K and is completed below 773 K depending on the heating rate. Hence, as the heating rate was increased, the reduction process gradually shifted to higher temperatures, i.e., the start and end of the reduction process occurs at higher temperature. The slope of the curves remains similar with changing heating rate. The mass loss recorded corresponds to the reduction of the surface oxide.

**Figure 2:**
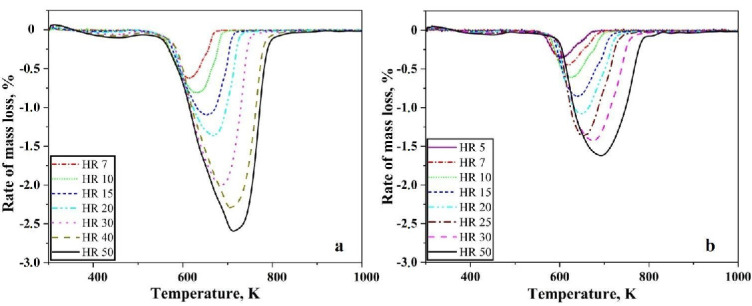
DTG curves for Fe nanopowder heated in pure hydrogen atmosphere, showing results at different heating rates in K/min for the sizes ranges (**a**) 40–60 nm, (**b**) 60–80 nm, respectively.

Figure 2a,b shows the first differential of the TG curves for reduction of oxide layer on iron nanopowder in the hydrogen at different heating rates for the size ranges of 40–60 nm and 60–80 nm, respectively. A single peak was observed in both the cases. The peak temperature was found to vary from 603 to 713 K for the 40–60 nm particle sizes with peak temperature being about 630 K for the heating rate of 7 K/min and about 713 K for 50 K/min. In the case of 60–80 nm particle size, the peak temperature varied from 599 to 685 K with about 600 K being the peak temperature for 5 K/min and about 684 for 50 K/min. The peak temperature of the DTG curves increases with increasing heating rate. Broadening of the peaks was observed as the heating rate increased. This peak temperature is used for calculating the apparent activation energy for the reduction reaction through Kissinger approach.

**Figure 3:**
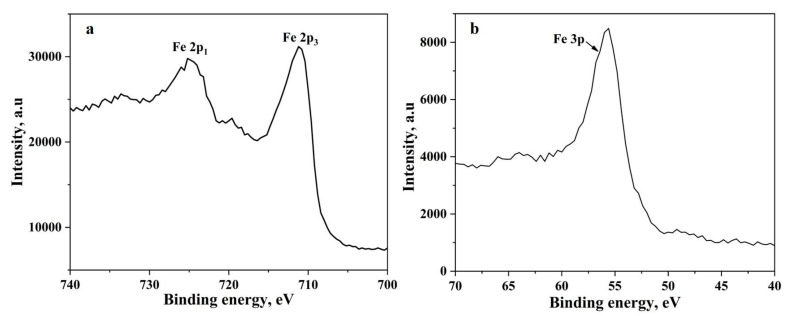
The XPS spectra of (**a**) Fe 2p peaks and (**b**) Fe 3p peak obtained from the iron nanopowder.

The metal particles are invariably covered by an oxide scale. The nanopowder particles are no exception to this fact. Metal nanopowder particles have core-shell structure, where the core is metal, and the shell is the oxide layer. The oxide scale on the metal particles must be reduced at least in part for the necks to form between them and subsequently for sintering to proceed. To understand the chemical constituents of the oxide scale techniques like X-ray photoelectron spectroscopy, Auger electron spectroscopy can be used. The thickness of the oxide scale can also be evaluated by applying different models using the thermogravimetric data obtained as above. For the case of water-atomized powder, the thickness has been assessed using XPS and has been found to be in the range of 4–7 nm [17]. The authors in their previous work have performed microstructural and surface characterization of the iron nanopowder used in the present study where the thickness of the oxide scale has been determined to be 3 nm [7]. The mass loss data in Figure 1 corresponds to the reduction of the above-mentioned oxide scale. Figure 3 shows that the oxide scale consists predominantly of Fe_2_O_3_. Yamashita et al. [18] have performed XPS investigations on Fe_2_O_3_ and Fe_3_O_4_ samples and found that the 2p_3_ peak appears at 711.0 eV and 3p peak appears at 55.6 eV for Fe_2_O_3_. The binding energies from literature correspond well with the peak positions obtained in the present study confirming that the oxide scale consists of Fe_2_O_3_. The TG and DTG curves show a single stage reduction of Fe_2_O_3_ to Fe.

#### 3.1.2. Reduction Kinetics for Removal of Oxide Layer on Fe Nanopowder

The reduction kinetics in this study has been addressed in terms of the extent of reaction or extent of conversion by a factor of conversion, α. The conversion fraction is obtained using Equation (3).

**Figure 4:**
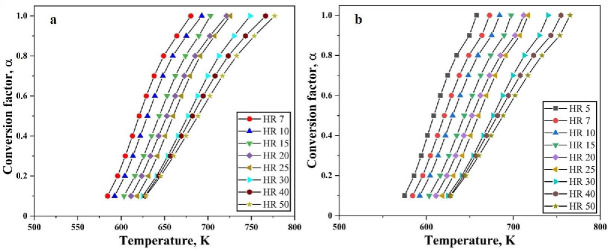
Variation of conversion factor as function of temperature at different heating rates for reduction of oxide film on Fe nanopowder of particle size ranges (**a**) 40–60 nm and (**b**) 60–80 nm, respectively.

Figure 4 shows the plots with varying degree of conversion for the reduction of the oxide film on the Fe nanopowder under hydrogen atmosphere for different heating rates against temperature. This is calculated based on the evaluation of conversion of the reduction reaction using Equation (3) and the temperature corresponding to the particular α, is taken from the TG curves. The total mass loss recorded from the TG is considered as complete reduction (100%) and total mass loss, in this case 5%, is taken as α = 1.0. Further calculations for different levels of conversion are done based on this.

There is no change in the behavior of the curves at different heating rates. The broadening of the peaks for higher heating rates is reflected in Figure 4 as the curves for higher heating rate have a broadening effect. As the reduction of the surface oxide is supposed to be a single stage process for Fe nanopowder, the slope appears basically the same.

**Figure 5:**
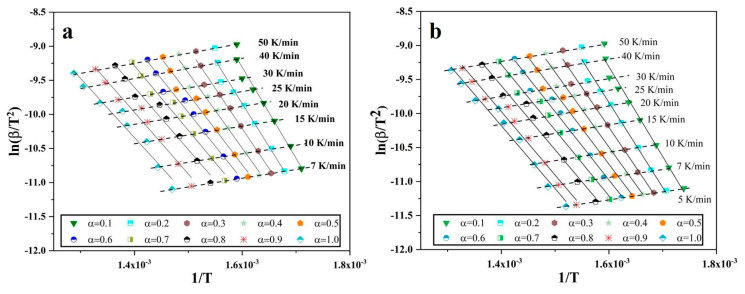
Plots of $\ln\frac{\beta }{T^{2}}$ vs. $\frac{1}{T}$ for different degree of conversion of oxide film and heating rates for Fe nanopowder of size ranges (**a**) 40–60 and (**b**) 60–80 nm, respectively.

It has already been established that the isoconversional methods for kinetics analysis use the exact temperature where a certain extent of conversion or reduction occur, and such exercise is iterated at different heating rates. The degree of conversion from 0.1 to 1.0 is investigated for the oxide film on Fe nanopowder, and from 0.03 to 1.0 for the reference Fe_2_O_3_ nanopowder. A plot is drawn using Equation (12) by utilizing the absolute temperature, degree of conversion for particular heating rate and multiple heating rate and degree of conversion data are then combined into a single plot (Figure 5). All the slopes in Figure 5a,b look similar at every condition of α.

**Figure 6:**
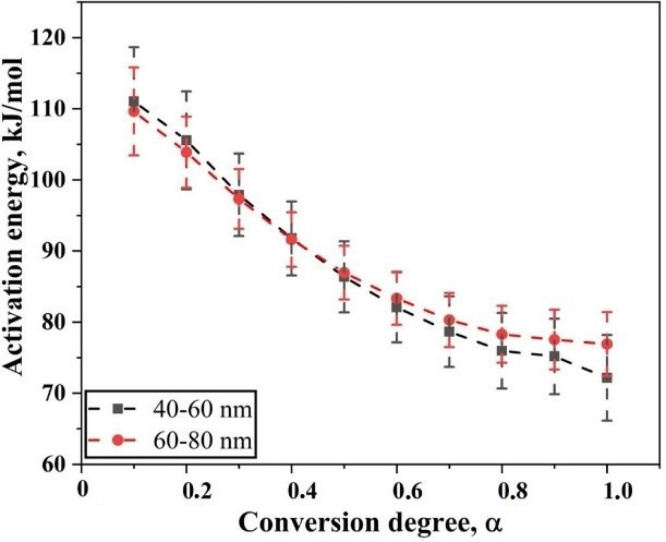
Plot showing the variation of apparent activation energy as the degree of conversion of the reduction of oxide film on Fe nanopowder of sizes 40–60 nm and 60–80 nm, respectively. The plot also indicates the scatter in the derived value of activation energy.

After plotting, least square linear fitting was applied to each set of points, where each set consists of 10 points that correspond to different heating rates for specific degree of conversion. The slope of the fit was taken that is $-\frac{E}{R}$, so the slope is then multiplied by −R to yield the value of apparent activation energy E. Through this way, the E was calculated for different degree of conversion, in the case of Fe nanopowder from 0.1 to 1.0. Figure 6 is the plot of the calculated activation energy at different degree of conversion.

The apparent activation energy for the reduction of surface oxide in Fe nanopowder for two different size grades varies from 65 to 118 and 71 to 115 kJ/mol for the 40–60 and 60–80 nm sizes of Fe nanopowder, respectively. Hence, an overall decrease in the value of apparent activation energy was observed as the reduction process proceeds. For both the size fractions of Fe nanopowder, the activation energy values were in the same range over varying degrees of conversion. From 0.1 < α < 0.7, the apparent activation energy values for both particle size ranges remained the same, while for the final stage of reduction, the values differed slightly.

### 3.2. Iron Oxide Nanopowder

#### 3.2.1. Non-Isothermal Reduction of Fe_2_O_3_ Nanopowder

**Figure 7:**
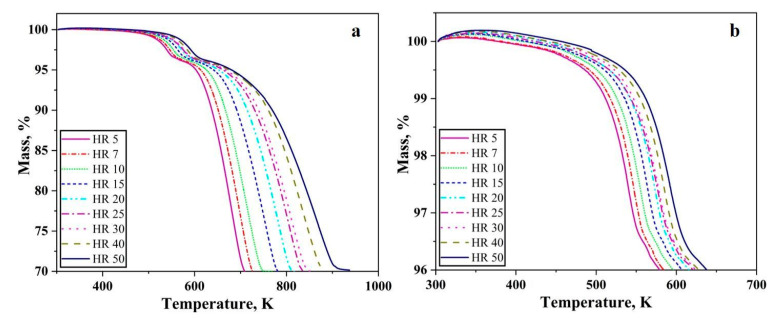
TG graphs of Fe_2_O_3_ nanopowder reduced in hydrogen atmosphere at various heating rates (**a**) till 973 K (**b**) till 623 K.

Figure 7 shows the TG curves of the Fe_2_O_3_ nanopowder under hydrogen atmosphere for the heating up to 923 K and 623 K at various heating rates.

The total mass loss of 30% was recorded at various heating rates. This mass loss corresponds well to the removal of oxygen from Fe_2_O_3_ in accordance with its composition, which essentially means that the reduction of Fe_2_O_3_ to Fe was complete at all conditions. As the heating rate increases, the TG curve gets broader. In other words, the reduction process is slowed down as the heating rate increases and attains a completion at higher temperatures. The start of the reduction process is also retarded with an increase in the heating rate. For a heating rate of 5 K/min, the complete reduction of the oxide finishes before the temperature reaches 723 K whereas in the case of 50 K/min complete reduction occurs around 923 K. Two different slopes were clearly observed from Figure 7a. Slope change is observed at around 3.5% mass loss or 96.5% remaining mass. For a heating rate of 5 K/min, the change in slope is observed at around 553 K whereas for 50 K/min it is 598 K (Figure 7b).

**Figure 8:**
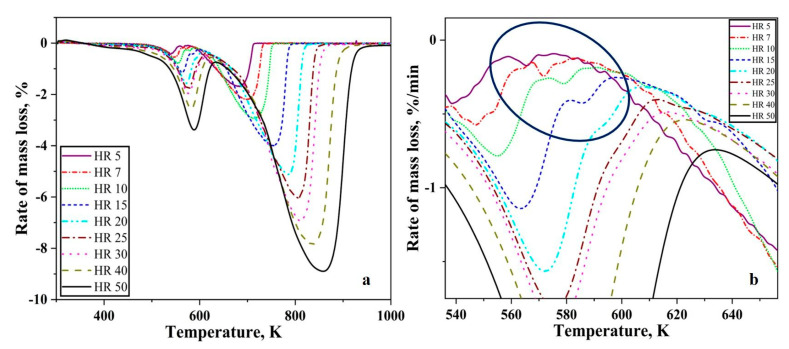
DTG curves of Fe_2_O_3_ nanopowder reduced in hydrogen atmosphere (**a**) complete curves, (**b**) showing a small peak in some of the DTG curves.

The slope change of TG curves can be interpreted or understood better when the first differential is taken. Figure 8 shows the DTG curve of Fe_2_O_3_ nanopowder reduced under hydrogen atmosphere at various heating rates. For all the heating rates the DTG curve is composed of two peaks namely, T1 and T2 (Figure 8a). These two peaks for all the heating rates occur in a temperature range of 533 < T1 < 593 K and 683 < T2 < 863 K. For heating rates 5, 7, 10, and 15 K/min, there is a small peak between observed between the first and second peaks (Figure 8b). This small peak merges with the first peak as the heating rate increases and it occurs in the temperature range of 563 to 603 K. There is a broadening of the second peak with increasing heating rate as the temperature to complete the reduction process also increases.

The slope of the TG curves in Figure 7a change as the temperature increases. For the convenience of understanding, the TG curve can be divided into two stages. The first stage corresponds to the reduction of Fe_2_O_3_ to Fe_3_O_4_ as a mass loss of around 3.5% which is close to the theoretical value of 3.34%. This is the prereduction step in the reduction of Fe_2_O_3_ to Fe prior to the second stage where the complete reduction to metal iron occurs. Therefore, the reduction process proceeds in two stages, Fe_2_O_3_ → Fe_3_O_4_ → Fe. This two-stage reduction agrees well with previous literature studies which report that the reduction proceeds in these two stages [9,19]. When reduced under isothermal conditions below 843 K, either Fe_2_O_3_ and Fe_3_O_4_ or Fe_3_O_4_ and Fe exist at any point throughout the experiment [20]. For the same amount of mass loss, at higher heating rates the residence time for the sample is reduced. Therefore, it must lose more mass during the reduced residence time. Hence, we see deeper peaks at higher heating rates. For the DTG curves, the peaks whose maxima is located between 533 and 593 K correspond to the reduction of Fe_2_O_3_ to Fe_3_O_4_ and the peaks whose maxima is located between 683 and 863 K correspond to the second reduction step from Fe_3_O_4_ to metallic iron.

**Figure 9:**
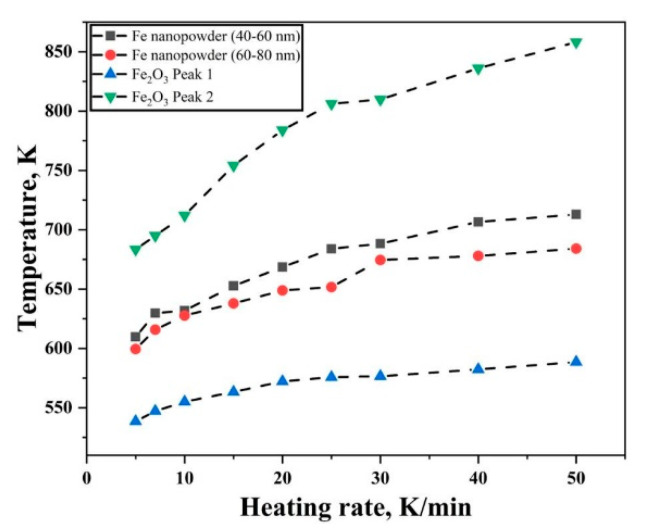
Peak temperature variation taken from DTG curves for reduction of oxide film on Fe nanopowder and reference Fe_2_O_3_ nanopowder.

The peak temperatures at different heating rates for Fe nanopowder and Fe_2_O_3_ nanopowder experiments are shown in Figure 9. The peak temperatures for Fe nanopowder lie in between the first and the second peaks of the Fe_2_O_3_ nanopowder. The scale is composed of Fe_2_O_3_ which is reduced in a single step to Fe. The peak temperature for reduction of the oxide film on Fe nanopowder is hence approximately an average of the peak values of the reference Fe_2_O_3_ nanopowder.

The oxide covering the iron nanopowder is reduced in a single step whereas the oxide nanopowder undergoes a two-step reduction process.

#### 3.2.2. Reduction Kinetics for Fe_2_O_3_ Nanopowder

**Figure 10:**
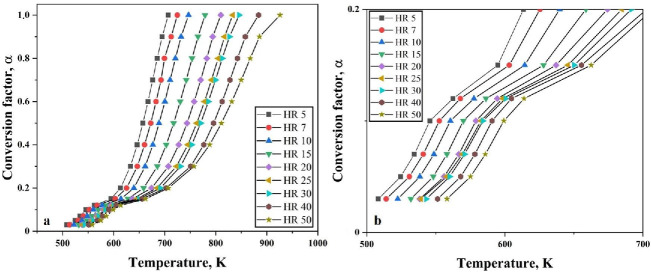
Variation observed in degree of conversion as function of temperature at varying heating rates for (**a**) complete heating range and (**b**) close-up view showing the slope change.

A total mass loss of 30% was observed when Fe_2_O_3_ nanopowder was reduced under hydrogen atmosphere in the TG. Hence, this total mass loss of 30% means α = 1.0. The degree of conversion is therefore taken at different stages of reduction namely, from 0.03 to 1.0. Figure 10 shows the changes in the degree of conversion as function of temperature at varying heating rates for Fe_2_O_3_ nanopowder reduced under hydrogen atmosphere. It can be clearly seen that the slope of the curves changes around α < 0.2. This is an indication that the nature of reaction is changed. Figure 10b shows that slope change is observed around α = 0.12. This slope change is in line with the change observed from TG curves where a slope change was observed at 3.5% mass loss.

The change in the slope of the curves at α = 0.12 or when the mass loss is 3.5% is attributed to the conversion of Fe_2_O_3_ to Fe_3_O_4_. Beyond this, the main reduction step, which is Fe_3_O_4_ to Fe commences. Similar studies carried out by Paurghahramani et al. [9] revealed both magnetite and hematite phases by means of X-ray analysis of the reduced products of sample heated to 450 °C. Change in the slope was also observed as the degree of conversion reached 0.11.

**Figure 11:**
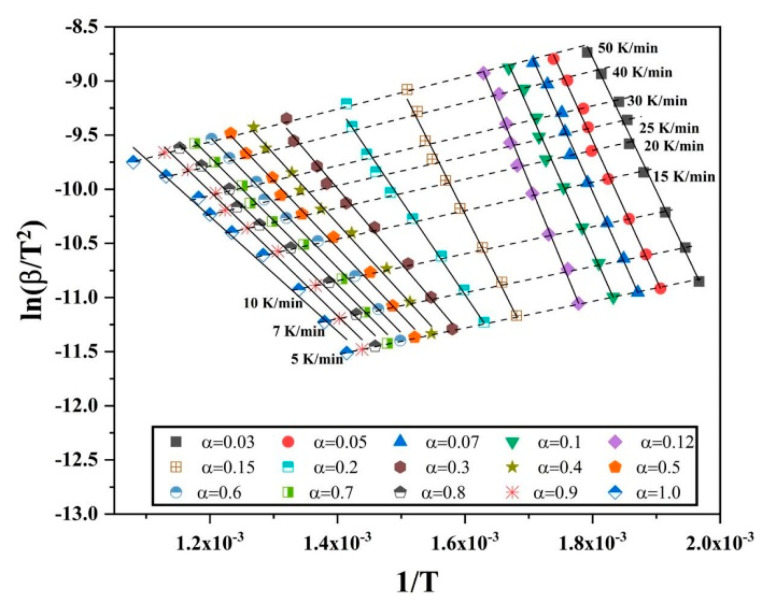
Iscoconversional plots of $\ln\frac{\beta }{T^{2}}$ vs. $\frac{1}{T}$ for different degrees of conversion when reducing Fe_2_O_3_ nanopowder in hydrogen.

The isoconversional plots ($\ln\frac{\beta }{T^{2}}$ vs. $\frac{1}{T}$) for different degrees of conversion of Fe_2_O_3_ are plotted in Figure 11. Similar to Figure 8, each set of points have been least-square fitted linearly and the slope was multiplied with -R to yield the apparent activation energy value for various values of α. The slope of the curves change as the value of α is increased. Beyond α = 0.12, a change is seen in the slope of the curves. For 0.3 < α < 1.0, the curves have a similar slope. This indicates a change in the reduction reaction.

**Figure 12:**
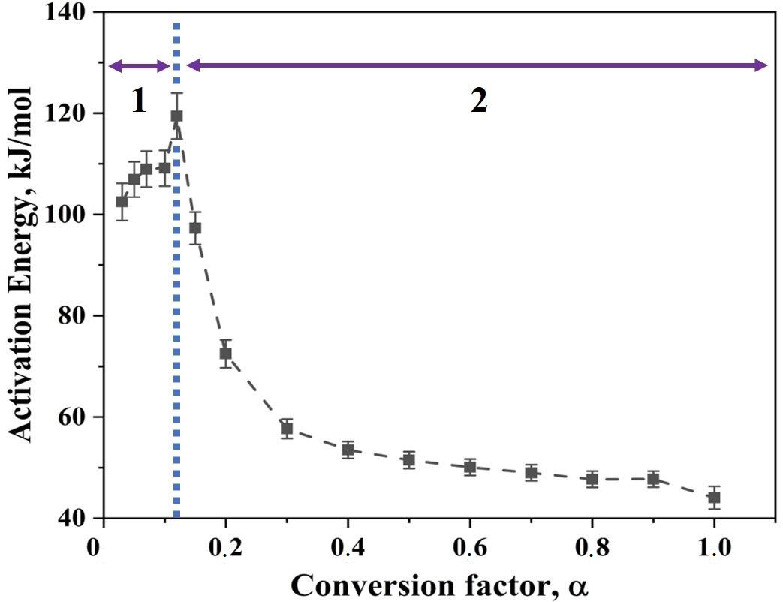
Plot depicting the variation in apparent activation energy as a function of conversion factor, α, for Fe_2_O_3_ nanopowder reduced in hydrogen where region 1 is the prereduction step whereas region 2 is the main reduction step.

**Figure 13:**
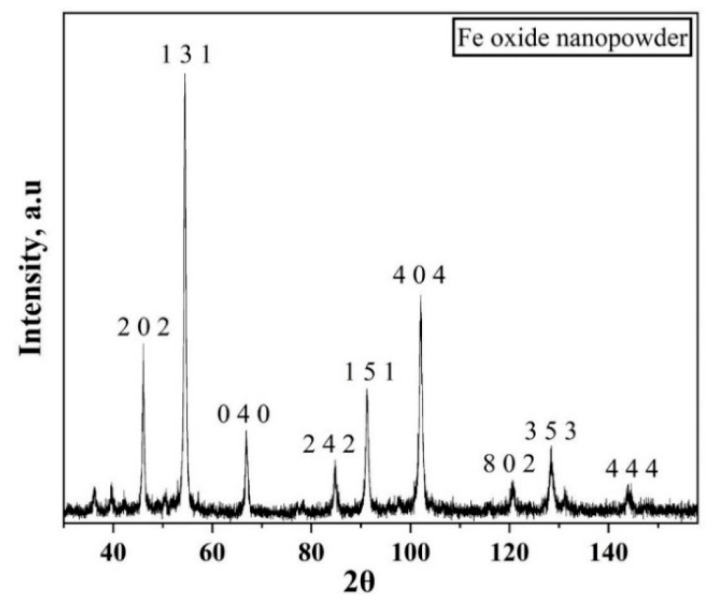
XRD pattern of iron oxide nanopowder showing the crystalline nature of the oxide used in the present study.

Figure 12 shows the variation in activation energy as the degree of conversion changes for Fe_2_O_3_ nanopowder reduced in hydrogen environment. The apparent activation energy for the reduction of Fe_2_O_3_ nanopowder in hydrogen environment varied from 45 to 120 kJ/mol. This variation in activation energy can be viewed as three distinct ranges according to the slope change. The initial range is 0.03 < α < 0.15, for which there is an increase in the apparent activation energy value from 105 to 120 kJ/mol. The second range, 0.15 < α < 0.4, is characterized by a drastic decrease in the value of apparent activation energy from 120 to 55 kJ/mol. For the third and final range, 0.4 < α < 1.0, the activation energy decreases steadily from 55 to 45 kJ/mol. It is known that the α < 0.15 corresponds to the reduction of Fe_2_O_3_ to Fe_3_O_4_. The later part of the conversion corresponds to the reduction reaction of Fe_3_O_4_ to Fe. The highest activation energy value in the initial range falls well within the broad range literature values of 75–245 kJ/mol for the reduction of Fe_2_O_3_ to Fe_3_O_4_. This reduction demands a higher activation energy in comparison to the second step of reduction which is the reduction of Fe_3_O_4_ to metallic Fe, for which values are between 15–172 kJ/mol have been quoted [19,21,22,23]. Though the value of activation energy is well within the reported range, the trend along the degree of reduction is different in the present case when compared to the reported data [23]. The iron oxide nanopowder used in the present study was crystalline in nature as determined from XRD (Figure 13). Furthermore, Scherrer analysis was performed and the crystallite size was calculated to be 25 nm. Table 1 lists activation energy values for the reduction of iron oxide.

### 3.3. Kissinger Approach for Iron and Iron Oxide Reduction Kinetics

**Figure 14:**
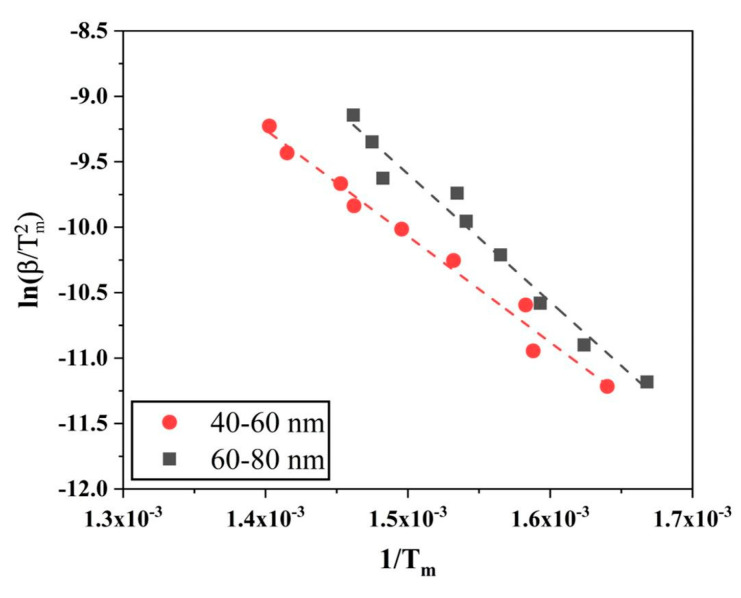
Kissinger plots to evaluate the activation energy for the reduction of surface oxide of Fe nanopowder of sizes (red) 40–60 and (black) 60–80 nm, respectively.

Figure 14 shows the Kissinger plots ($\ln\left(\frac{\beta }{T_{m}^{2}}\right)$ versus $\frac{1}{T_{m}}$) for reduction of the oxide film on Fe nanopowder of the two different size fractions 40–60 and 60–80 nm, respectively. The slope of the curve was multiplied with -R and the activation energy values were caculated to be 67.5 ± 5.0 and 81.3 ± 3.4 kJ/mol for the 40–60 and 60–80 nm particles, respectively. These values lie close to the activation energy values obtained from the isoconversional method for α = 1.0.

**Figure 15:**
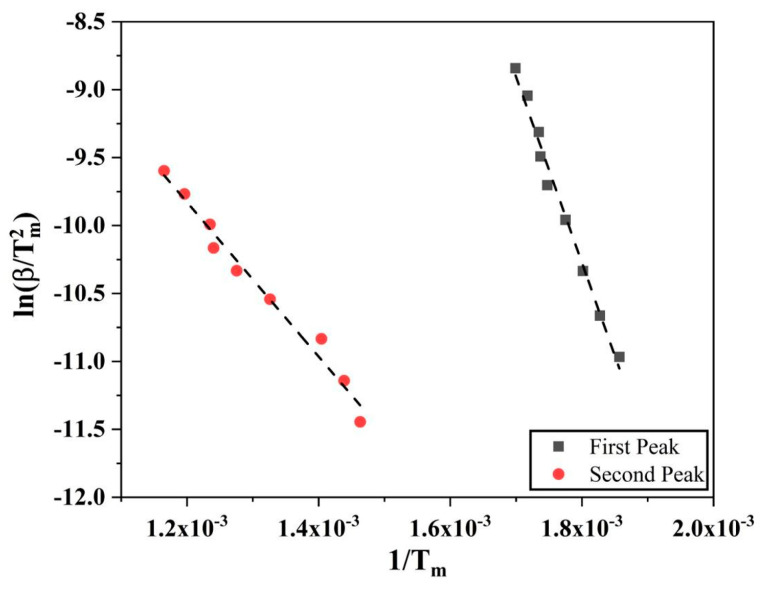
Kissinger plots for the Fe_2_O_3_ to Fe_3_O_4_ and Fe_3_O_4_ to Fe reduction reactions.

Similar calculations using Kissinger method were done for reduction of Fe_2_O_3_ in hydrogen as well. As the reduction of Fe_2_O_3_ to Fe is a two-stage process, two different peaks from the DTG graphs (Figure 8) were used and processed for the activation energy values. Figure 15 shows the Kissinger plots for both the first and second peaks of reduction of Fe_2_O_3_. The activation energy for the first reduction step, Fe_2_O_3_ to Fe_3_O_4_, was calculated to be 113.7 ± 4.9 kJ/mol, whereas for the second step, Fe_3_O_4_ to Fe, was calculated to be 47.2 ± 2.5 kJ/mol.

The obtained values from Kissinger method for activation energy were in the range of values calculated using isoconversional approach. A comparison is shown in Table 2.

**Figure 16:**
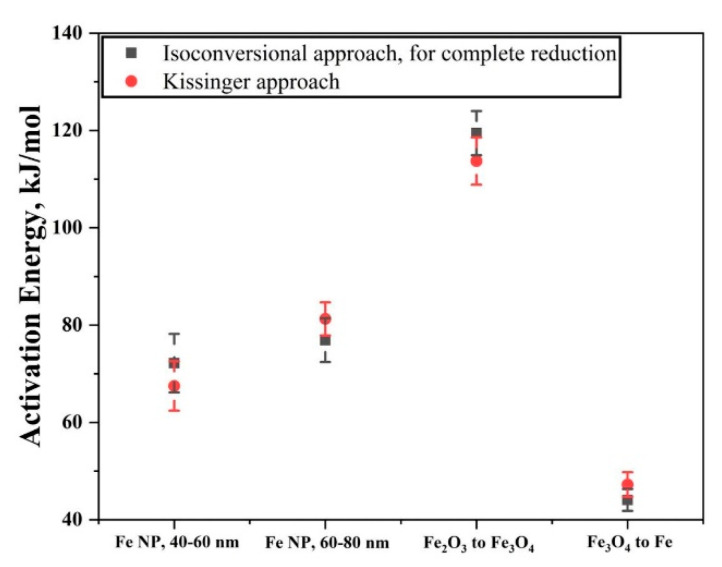
Plot showing the activation energy obtained using isoconversional approach, for full reduction reaction and values obtained using Kissinger approach.

Figure 16 shows a plot comparing the activation energy values using two different approaches. For the isoconversional approach, the values of apparent activation energy were taken when the reduction reaction was complete. The values obtained using two different approaches overlap and are in the range of values from other studies. Although the values obtained using Kissinger method correspond well with the values obtained from isoconversional method for complete reduction, Kissinger method takes absolute temperature values into account. Therefore, it provides a single value for the reduction process. This does not account for the varying activation values as the reaction proceeds and it is the isoconversional approach which offers this in detail.

### 3.4. Proposed Model

It is generally accepted that the reduction of iron oxide (Fe_2_O_3_), in its bulk form, proceeds via a two-step process to convert to metallic Fe. The same was observed even for the powder and particulate forms. For the reaction where iron oxide is reduced to metallic iron, the mechanism followed depends on the conditions chosen for the analysis [25]. For particulate materials, in the case of coarse particles (millimeter size range), reduction reportedly proceeds via a phase boundary mechanism or topochemical mode of reaction. For finer particles/powder, however, a different reaction mechanism is seen to be operative, particularly at lower temperatures, which is nucleation and growth [26].

In the current study it has been corroborated that even in the nanometer regime, a two-step process is involved in the reduction of iron oxide for the case of nanopowder. For the case of iron nanopowder, a single step reaction is however shown to occur for the reduction of surface iron oxide to metallic Fe.

**Figure 17:**
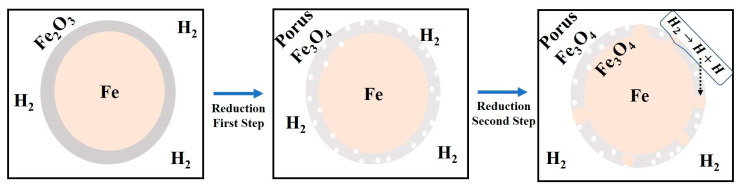
Proposed model for the reduction of surface oxide on iron nanopowder.

In this regard, a qualitative model is proposed to further explain the reduction of the surface oxide of iron nanopowder based on the knowledge developed from the reaction kinetics of iron oxide nanopowder (Figure 17) where the oxide layer is assumed to consist of Fe_2_O_3_ (Figure 3). The densities of the oxide layers being 5.2 and 5.7 g/cm^3^ for Fe_2_O_3_ and Fe_3_O_4_ respectively. The model is based on following assumed conditions:

- During the prereduction step, Fe_2_O_3_ is reduced to Fe_3_O_4_ completely. As the density of Fe_3_O_4_ is higher than that of Fe_2_O_3_, a porous Fe_3_O_4_ structure is expected upon transformation.
- The porous Fe_3_O_4_ is reduced to metallic Fe during the main reduction step, resulting in islands of metallic Fe.
- Catalytic reaction: The freshly formed metallic iron surfaces exhibit an auto-catalytic nature, supporting the chemisorption and disassociation of hydrogen molecules to yield active hydrogen atoms which are transferred or transported from metal surface to the metal/oxide interfaces through ‘portholes’ of water vapour [25,27].

Through the above conditions a to c, the metal surface is created early on and reactions can proceed in parallel when having oxide film on Fe nanopowder, whereby there will be no clear differentiation of the two reduction stages as observed for the Fe_2_O_3_ reference nanopowder.

## 4. Conclusions

The present study addressed the reaction kinetics for the reduction of oxide film on iron nanopowder in hydrogen, including the comparison with the reduction of Fe_2_O_3_ nanopowder as reference under same conditions. The experiments were done using high sensitivity thermogravimetric analysis and two size ranges of 40–60 nm and 60–80 nm of the iron nanopowder were used. The mass loss of about 5% was recorded for both size fractions of iron nanopowder for all the heating rates up to 50 K/min. This mass loss corresponds to the complete reduction of the surface oxide, supposed to be mainly Fe_2_O_3_. The mass loss curve showed a single maximum peak, hence indicating that the surface oxide is reduced to metallic Fe in a single stage process. The calculated activation energy from the TG measurements using both isoconversional and Kissinger approach was in the range of the reported data for the reduction of the iron oxide in hydrogen. The TG and DTG analysis of Fe_2_O_3_ revealed instead that the reduction of Fe_2_O_3_ to metallic Fe followed a two-stage process involving first the decomposition Fe_2_O_3_ → Fe_3_O_4_ and then the final reduction Fe_3_O_4_ → Fe. For the initial range of α, 0.03 < α < 0.15, the activation energy was in the range of 105 to 120 kJ/mol. This corresponds to the reduction of Fe_2_O_3_ → Fe_3_O_4_ in hydrogen. A drastic reduction in the value of activation energy ranging from 120 to 45 kJ/mol, was observed for α > 0.15 which corresponded the reduction stage Fe_3_O_4_ → Fe. The decreasing trend could be explained using the nucleation and growth model where the activation energy needed for nucleation is higher than needed for growth. The activation energy values fall in the range of reported data for the reduction of iron oxide in hydrogen. The reduced activation energy can be attributed to the catalytic activity of reduced Fe particles which facilitate the reduction of adjacent oxide particles. In conclusion, it can be said that the surface iron oxide is reduced to metallic Fe particles and the newly formed metallic Fe surface accelerates the reduction process by serving as an autocatalytic surface.

## Figures and Tables

**Table 1:** Apparent activation energy of iron oxide reduction (kJ/mol).

Iron Oxide	Activation Energy, kJ/mol	Reduction	Temperature Range, °C
Granulated Fe_2_O_3_ nanopowder [23] α-Fe_2_O_3_ → Fe_2_O_3_ → Fe	75–125	Hydrogen reduction, Isoconversional approach	320–500
Ball milled Fe_2_O_3_ nanopowder agglomerates [21] α-Fe_2_O_3_ → Fe_2_O_3_ → Fe	20–46	Hygrometry method, Hydrogen reduction Isoconversional approach	270–580
Both natural and laboratory iron oxide α-Fe_2_O_3_ →Fe_3_O_4_ [24]	108	Hydrogen reduction	250–400
99.8% pure iron oxide α-Fe_2_O_3_ → Fe_3_O_4,_ [19]	76	Hydrogen reduction	220–683
α-Fe_2_O_3_ → Fe_3_O_4_, [19]	95	H_2_–N_2_	337–604
α-Fe_2_O_3_ → Fe_3_O_4_, [19]	114	CO	265–482
Fe_3_O_4_ → Fe, [19]	39–88	Hydrogen reduction	220–683
Fe_3_O_4_ → Fe, [19]	36–103	H_2_–N_2_	337–604
Fe_3_O_4_ → Fe, [19]	40–114	CO	265–482
α-Fe_2_O_3_ →Fe_3_O_4_, [22]	90	Temperature programmed reduction	230–380
Fe_3_O_4_ →Fe, [22]	70	Temperature programmed reduction	330–730
Iron oxide nanopowder α-Fe_2_O_3_ → Fe_3_O_4_, this work	105–120	Hydrogen reduction Isoconversional approach	230–430
Fe_3_O_4_ → Fe, this work	45–55	Hydrogen reduction Isoconversional approach	430–680

**Table 2:** Activation energy values for different reduction reactions using two different approaches.

Reaction	Activation Energy, Isoconversional Approach, kJ/mol	Activation Energy, Kissinger Approach, kJ/mol
Fe nanopowder surface oxide reduction (40–60 nm)	118–65	62–70
Fe nanopowder surface oxide reduction (60–80 nm)	115–71	78–84
Fe_2_O_3_ nanopowder, Fe_2_O_3_ → Fe_3_O_4_	105–120	109–117
Fe_2_O_3_ nanopowder, Fe_3_O_4_ → Fe	55–45	45–55

## Data Statement

The raw/processed data required to reproduce these findings cannot be shared at this time as the data also forms part of an ongoing study.
